# An efficient deep learning strategy for accurate and automated detection of breast tumors in ultrasound image datasets

**DOI:** 10.3389/fonc.2024.1461542

**Published:** 2025-03-03

**Authors:** Luyao Li, Yupeng Niu, Fa Tian, Bin Huang

**Affiliations:** ^1^ Department of Ultrasound, Zhejiang Hospital, Hangzhou, China; ^2^ College of Information Engineering, Sichuan Agricultural University, Ya’ an, China

**Keywords:** breast cancer detection, ultrasound imaging, deep learning, U2NET-Lite, DeepCardinal-50

## Abstract

**Background:**

Breast cancer ranks as one of the leading malignant tumors among women worldwide in terms of incidence and mortality. Ultrasound examination is a critical method for breast cancer screening and diagnosis in China. However, conventional breast ultrasound examinations are time-consuming and labor-intensive, necessitating the development of automated and efficient detection models.

**Methods:**

We developed a novel approach based on an improved deep learning model for the intelligent auxiliary diagnosis of breast tumors. Combining an optimized U2NET-Lite model with the efficient DeepCardinal-50 model, this method demonstrates superior accuracy and efficiency in the precise segmentation and classification of breast ultrasound images compared to traditional deep learning models such as ResNet and AlexNet.

**Results:**

Our proposed model demonstrated exceptional performance in experimental test sets. For segmentation, the U2NET-Lite model processed breast cancer images with an accuracy of 0.9702, a recall of 0.7961, and an IoU of 0.7063. In classification, the DeepCardinal-50 model excelled, achieving higher accuracy and AUC values compared to other models. Specifically, ResNet-50 achieved accuracies of 0.78 for benign, 0.67 for malignant, and 0.73 for normal cases, while DeepCardinal-50 achieved 0.76, 0.63, and 0.90 respectively. These results highlight our model’s superior capability in breast tumor identification and classification.

**Conclusion:**

The automatic detection of benign and malignant breast tumors using deep learning can rapidly and accurately identify breast tumor types at an early stage, which is crucial for the early diagnosis and treatment of malignant breast tumors.

## 1 Introduction

Breast cancer is regarded as the second common cancer globally after lung cancer, the fifth common reason for cancer death (1). It is critical to detect breast cancer at an early stage in order to reduce the mortality rate (2). Many imaging tools are available for prior identification and early treatment of breast cancer. However, ultrasound is noninvasive, well tolerated by women, and radiation free; therefore, it is commonly used in the diagnosis of breast tumors (3).

Nevertheless, manual ultrasound breast cancer diagnosis takes a long time and requires an experienced physician to make a relatively accurate judgment, so the development of an effective automated system for early detection of breast cancer is of great clinical interest.

With the rise of artificial intelligence technology, deep learning for breast ultrasound detection has been increasingly studied. Yuan Xu et al. (4), have introduced their machine learning based work of medical BUS images’ segmentation, proposing a CNNs based fully automatic BUS images’ segmentation method into four major tissues: skin, fibroglandular tissue, mass, and fatty tissue, resulting in efficient automated segmentation providing a helpful reference to radiologists for better breast cancer characterization and breast density assessments. Y. Lei et al. (5), have introduced their study for breast tumor segmentation in three dimensional (3D) ABUS, proposing a developed Mask scoring region-based CNN (Mask R-CNN) consists of five subnetworks: a backbone, a regional proposal network, a region CNN head, a mask head, and a mask score head. Their approach has been validated on 70 patients’ images with ground truth manual contour, resulting in an efficient segmentation of breast cancer’s volume from ABUS images. Byra (6) introduced a deep learning-based framework for the classification of breast mass from ultrasound images. They used transfer learning (TL) and added deep representation scaling (DRS) layers between pre-trained CNN blocks to improve information flow. Only the parameters of the DRS layers were updated during network training to modify the pre-trained CNN to analyze breast mass classification from the input images. The results showed that the DRS method was significantly better compared with the recent techniques. Kiran Jabeen et al. (7) proposes a new framework for breast cancer classification from ultrasound images that employs deep learning and the fusion of the best selected features. The proposed framework is divided into five major steps, the experiment was conducted on an augmented Breast Ultrasound Images (BUSI) dataset, and the best accuracy was 99.1%. When compared with recent techniques, the proposed framework outperforms them.

**Figure 1:**
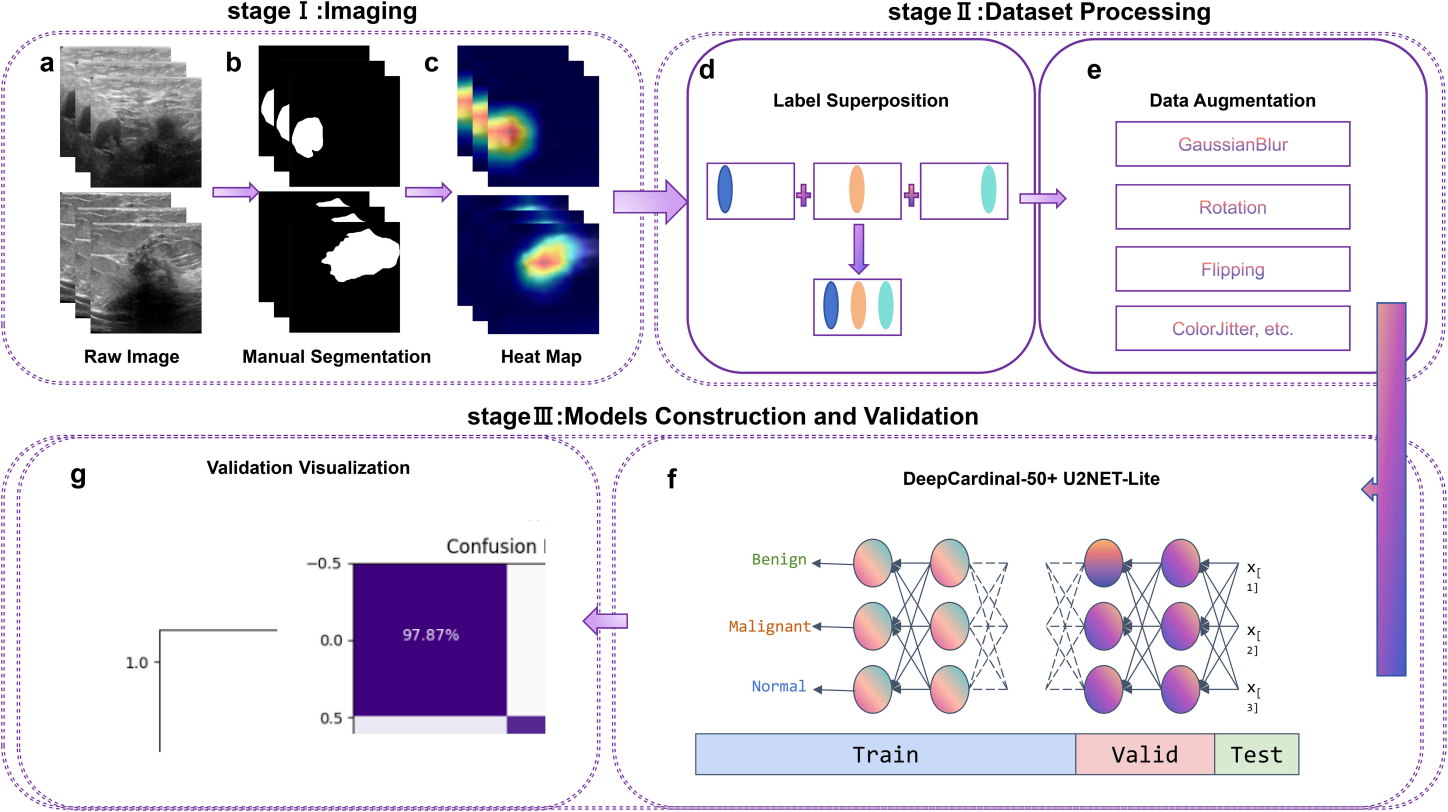
Workflow, including Stage I (Imaging), Stage II (Dataset Processing), and Stage III (Model Construction and Validation). Labels **(A–G)** correspond to different processing steps, as indicated in the figure.

In this study, we present an intelligent and precise auxiliary diagnosis method for distinguishing between benign and malignant breast tumors, based on machine learning and ultrasonography. This research employs the DeepCardinal-50 and U2NET-Lite deep learning models, combined with high-precision processing of breast ultrasound images, to enhance the accuracy and efficiency of breast cancer detection. The optimized U2NET-Lite model improves real-time performance, while DeepCardinal-50 enhances the processing capability for complex images. The detailed research methodology and workflow are illustrated in **Figure 1**.

## 2 Methods

### 2.1 Data acquisition

**Figure 2:**
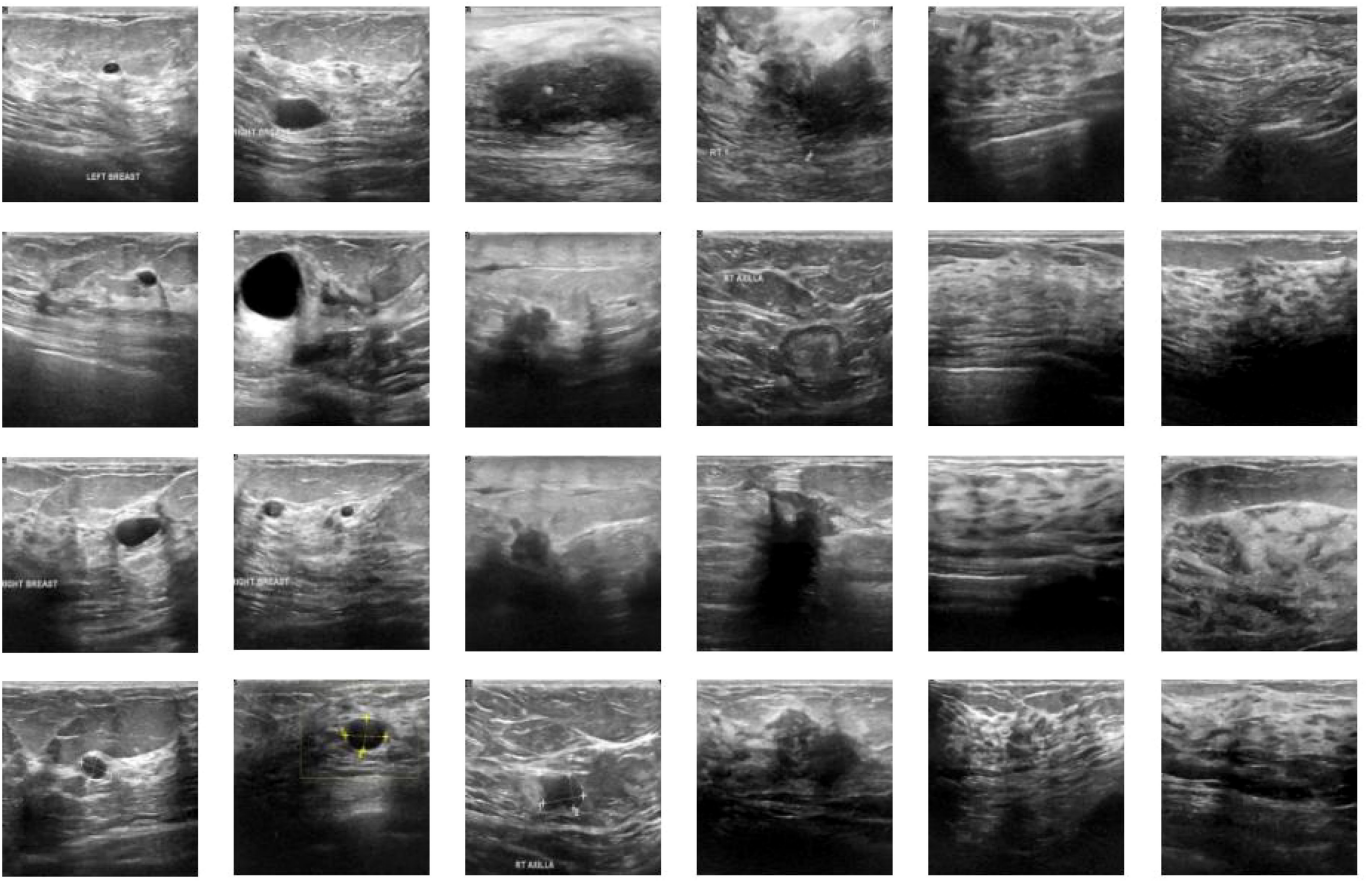
Dataset visualization.

We utilized breast ultrasound images from women aged 25 to 75, collected in 2018 (AI-Dhabyani W, Gomaa M, Khaled H, Fahmy A. Dataset of breast ultrasound images. Data in Brief. 2020 Feb;28:104863. DOI: 10.1016/j.dib.2019.104863). The dataset includes 600 female patients, comprising 780 images with an average size of 500x500 pixels, in PNG format. Ground truth images are presented alongside the original images. The images are categorized into three classes: normal, benign, and malignant. **Figure 2** shows a portion of our dataset.

### 2.2 Data preprocessing

In this study, label overlay plays a pivotal role in data preprocessing. By seamlessly integrating precise pathological classification labels into the ultrasound images, this approach aims to enhance the model’s capacity to recognize intricate features and effectively distinguish between benign and malignant breast tumors. Unlike conventional methods, this label overlay technique embeds specific pathological information directly into the visual data, creating a richer and more informative dataset. A more detailed explanation of its implementation would further illustrate its uniqueness. Specifically, describing how the overlay process is executed, including the technical workflow and challenges addressed, would provide deeper insights. This innovative preprocessing step not only ensures that the model benefits from enriched training data but also directly contributes to improved diagnostic accuracy by facilitating more precise tumor identification and classification.

During the image cleaning and optimization phase, we applied a series of processes to the breast ultrasound images, including noise removal, filtering, and contrast adjustment. These steps are intended to eliminate interference, enhance important features, and improve overall image quality. Noise removal helps reduce misleading information, while filtering and contrast adjustment highlight critical pathological features in the images.

In this study, data augmentation is another key step to enhance the model’s generalization capability and reduce the potential for overfitting. The data augmentation techniques applied include random horizontal flipping, vertical flipping, random rotation, color jittering, and Gaussian blur. These methods introduce diverse image transformations, simulating different shooting conditions and background variations, thus ensuring that the model maintains high accuracy and robustness in variable real-world application scenarios. Additionally, these techniques help balance the dataset, particularly when certain types of images are less abundant, reducing the risk of the model being biased towards a specific category.

### 2.3 Construction of focal segmentation model

The lesion segmentation model in this study is built upon an advanced deep learning architecture, specifically the custom-designed U2NET-Lite model. The U2NET-Lite model is notable for its lightweight design, characterized by reduced network depth and fewer parameters, making it well-suited for the real-time processing requirements of breast ultrasound images. This model is highly efficient in extracting deep and high-resolution features, particularly through the use of a double nested U-structure and Residual U-blocks (RSU) to achieve multi-scale feature fusion, enhancing the ability to identify and segment target objects. The total model size of U2NET-Lite is 4.7 MB, significantly smaller than the standard U2NET version’s 176.3 MB, making it more suitable for resource-constrained environments.

**Figure 3:**
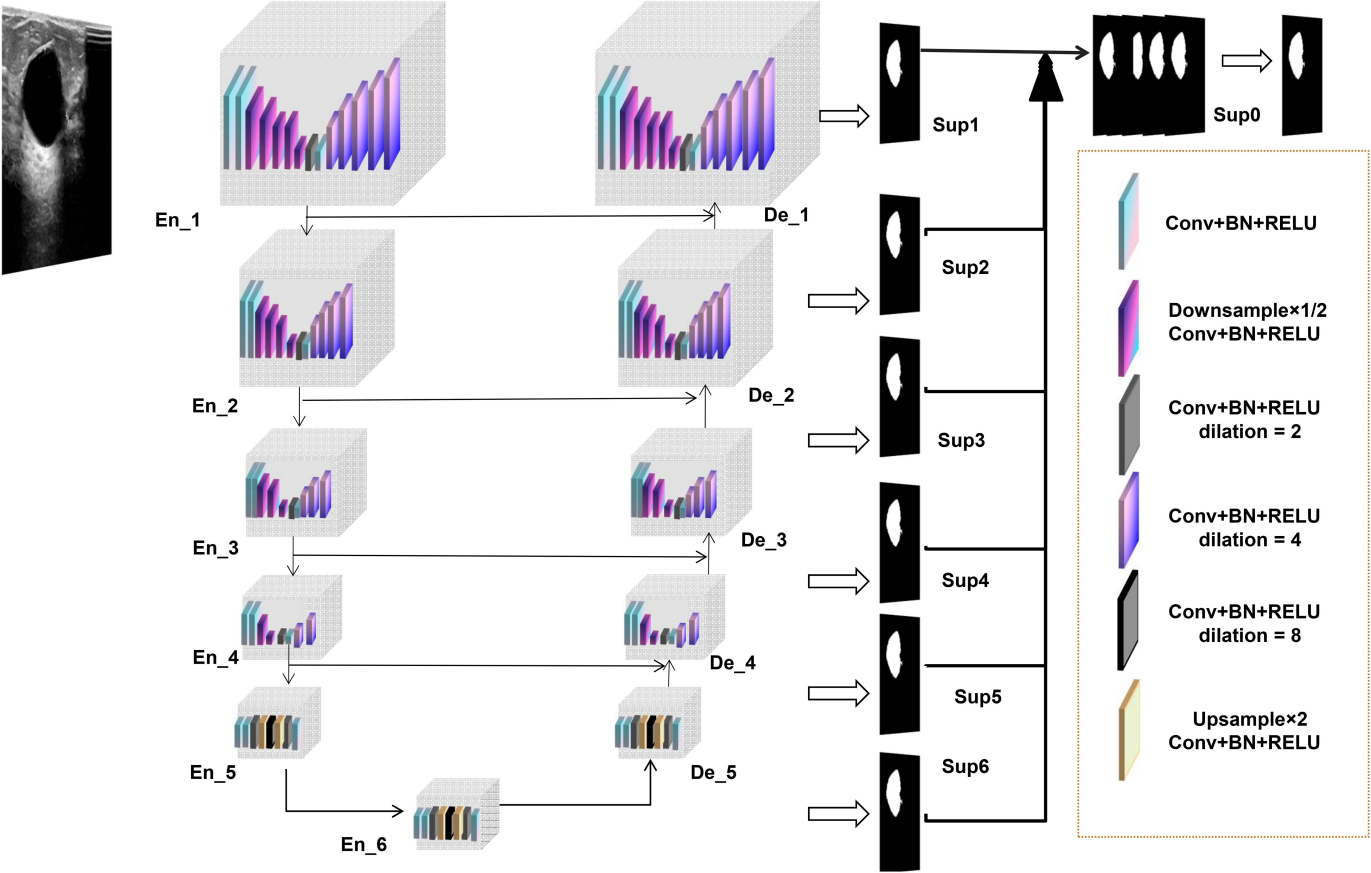
U2NET-Lite Network Architecture Diagram.

Through its lightweight design and multi-scale feature fusion, U2NET-Lite maintains high accuracy and efficiency in the task of ultrasound segmentation of breast tumors. **Figure 3** illustrates our model’s network architecture.

### 2.4 Construct diagnostic model

#### 2.4.1 Proposed deep learning-based model

In the application of deep learning for breast tumor recognition, researchers have proposed and validated numerous models, with ResNet and AlexNet being among the most common. These models have achieved certain successes in the automatic identification and classification of breast tumors, demonstrating the immense potential of deep learning in medical imaging. However, traditional models face some limitations when processing specific breast ultrasound images. For instance, while ResNet addresses the vanishing gradient problem in deep network training through residual connections, it is not highly efficient in handling high-resolution and complex breast ultrasound images, often leading to computational burdens (8). AlexNet, as a pioneer in convolutional neural networks, has achieved significant results in some image classification tasks, but its shallow structure is inadequate for dealing with the complexity and high-resolution demands of breast ultrasound imaging (9). Moreover, these models fall short in real-time performance and lightweight design.

**Figure 4:**
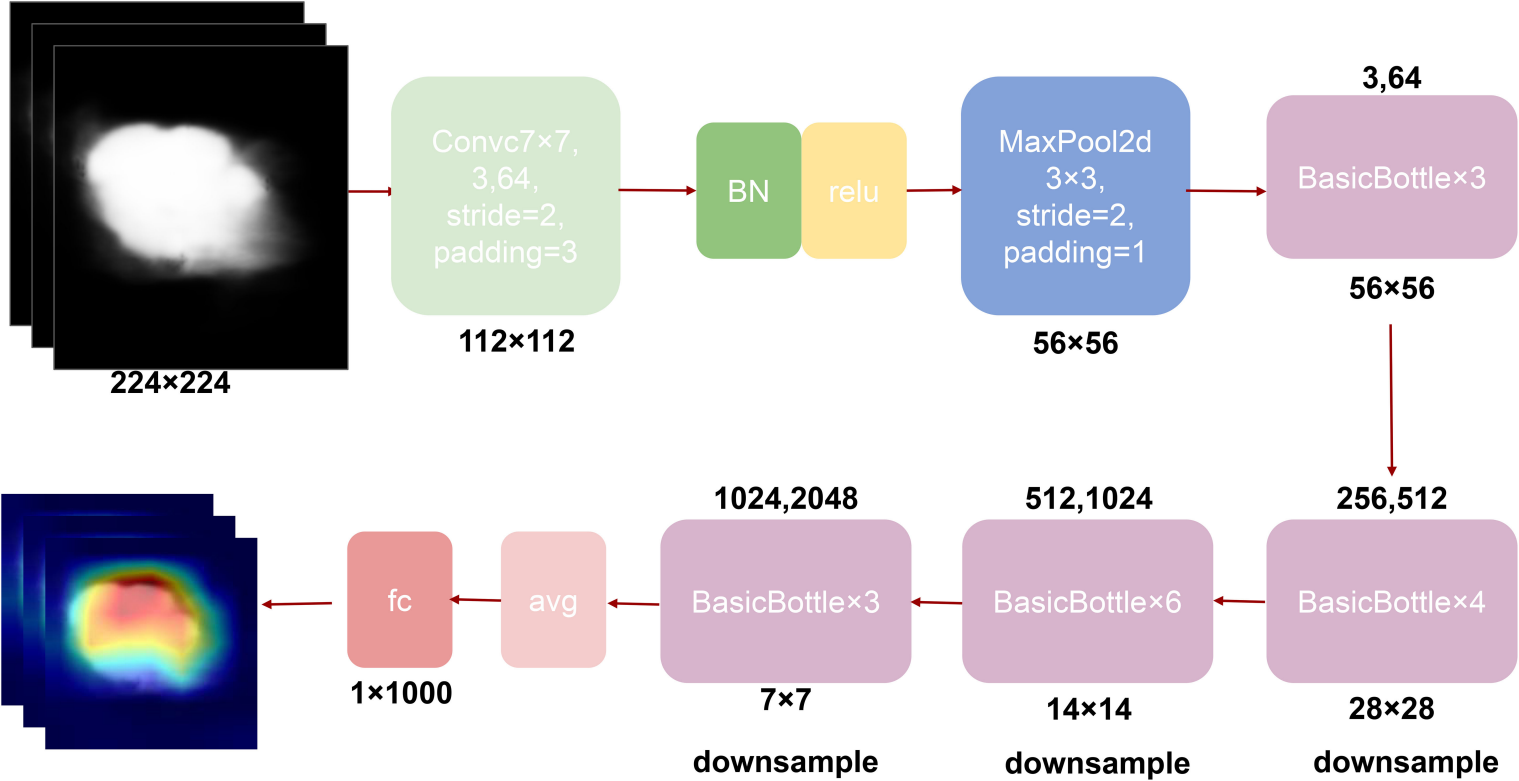
DeepCardinal-50 Model.

To overcome these limitations, our study proposes the DeepCardinal-50 model, which employs an improved residual network architecture, particularly emphasizing cardinality and the application of grouped convolutions. This model uses more convolutional kernels in the first two layers of each block, with 32 groups and 4 channels per group, significantly increasing the network’s width and capacity. The detailed network structure is shown in **Figure 4**. This design not only enhances the model’s learning ability but also maintains low latency and computational cost. Additionally, while achieving high performance, the size of the DeepCardinal-50 model is comparable to ResNet-50, yet its performance is equivalent to that of the much deeper ResNet-101 model.

Our model, through the innovative design of the aforementioned architecture, demonstrates several significant advantages. The DeepCardinal-50, with its optimized residual network structure, exhibits outstanding performance in classification tasks. This design not only enhances the model’s accuracy and efficiency but also makes it suitable for clinical application scenarios, meeting the requirements for real-time performance and lightweight deployment.

#### 2.4.2 Comparative models

ResNet (Residual Network) is a popular deep learning architecture commonly used for image recognition and classification tasks (8). In the field of breast tumor identification, ResNet addresses the training challenges of deep networks by introducing residual connections, allowing the network to be deeper and more effectively trained, thereby improving classification accuracy. AlexNet is a milestone model in deep learning, having a profound impact on image classification tasks (10). Despite its relatively simple network structure, AlexNet demonstrated the potential of deep convolutional networks for processing medical images, laying the groundwork for subsequent research. The U2NET model, particularly its lightweight version U2NET-Lite, excels in breast tumor detection due to its efficient deep and high-resolution feature extraction capabilities (11). Its double nested U-structure and Residual U-block (RSU) design optimize image semantic segmentation, making it especially suitable for handling detail-rich medical images (12–15).

### 2.5 Experimental setup

For the DeepCardinal-50 model, the architecture employed 32 groups with 4 channels per group in the grouped convolutions, significantly enhancing the feature extraction capability. The model was trained using the Adam optimizer with an initial learning rate of 0.0001, accompanied by a learning rate scheduler that reduced the rate by a factor of 0.1 after 10 epochs of no improvement in validation loss. A batch size of 16 was utilized, and training was conducted over 100 epochs. To optimize the binary classification task, the cross-entropy loss function was employed, ensuring stable and efficient learning throughout the training process.

For the U2NET-Lite model, a lightweight network architecture with a double nested U-structure and Residual U-blocks (RSU) enabled multi-scale feature fusion, which was critical for precise segmentation. This model was trained with a batch size of 8 using the SGD optimizer, configured with a momentum of 0.9 and a learning rate of 0.001. To mitigate overfitting, a weight decay of 0.0005 was applied, and the segmentation task was optimized using the Dice loss function. The experimental setup included datasets divided into a 12:3 ratio for segmentation and an 11:1:3 ratio for classification (training, validation, and testing). These divisions ensured a balanced dataset allocation for robust model evaluation. Experiments were executed in a Python 3.8 environment on an Ubuntu 20.04 system, with PyTorch 1.10.0 and CUDA 11.3 for computational acceleration. The server infrastructure included RTX 4090 GPUs and 15 vCPUs of an Intel Xeon Platinum 8358P CPU @ 2.60GHz, providing the computational resources necessary for high-performance experimentation.

### 2.6 Model evaluation

When evaluating deep learning models, we commonly use several key metrics to measure performance, including accuracy (ACC), F1 score, and Area Under the Curve (AUC). These evaluation metrics collectively describe the model’s performance in various aspects, such as prediction accuracy, comprehensiveness, and the consistency between predicted and actual results. First, accuracy (ACC) is the most straightforward evaluation metric. It is the ratio of correctly classified samples to the total number of samples. The mathematical formula is:


(1) $$ACC=\frac{\left(TP+TN\right)}{\left(TP+TN+FP+FN\right)}$$

where TP represents the number of true positive samples, TN represents the number of true negative samples, FP represents the number of false positive samples, and FN represents the number of false negative samples. Higher accuracy indicates a more effective classifier with more precise predictions.

Second, the F1 score is the harmonic mean of precision and recall. Precision represents the number of samples correctly identified as positive; recall represents the proportion of actual positive samples correctly predicted. The F1 score is calculated as:


(2) $$PRE=\frac{TP}{\left(TP+FP\right)}$$

(3) $$REC=\frac{TP}{\left(TP+FN\right)}$$

(4) $$F=\frac{(\alpha^{2}+1)P*R}{\alpha^{2}(P+R)}$$

When α=1\beta = 1α=1, it is the common F1 score.


(5) $$F_{1}=\frac{2P*R}{P+R}$$

Lastly, the Area Under the Curve (AUC) is a critical metric for evaluating model prediction performance. The larger the area under the ROC curve, the better the model’s predictive performance.

These metrics provide a comprehensive evaluation of the model’s effectiveness in different aspects, ensuring a robust assessment of its performance.

## 3 Results

### 3.1 Segmentation model results

In this study, we evaluated the performance of the U2NET-Lite model in the task of breast tumor segmentation. The results demonstrate that the model exhibits excellent performance in processing breast ultrasound images. In the experimental test set, U2NET-Lite achieved an accuracy of 97%, indicating its high precision in distinguishing tumors from normal tissue. The model’s recall rate reached 0.7961, showing its effectiveness in capturing abnormal regions in breast ultrasound images. Notably, the model’s Intersection over Union (IoU) was 0.7063, further proving U2NET-Lite’s strong capability in image segmentation tasks, accurately identifying and segmenting breast tumor areas.

**Figure 5:**
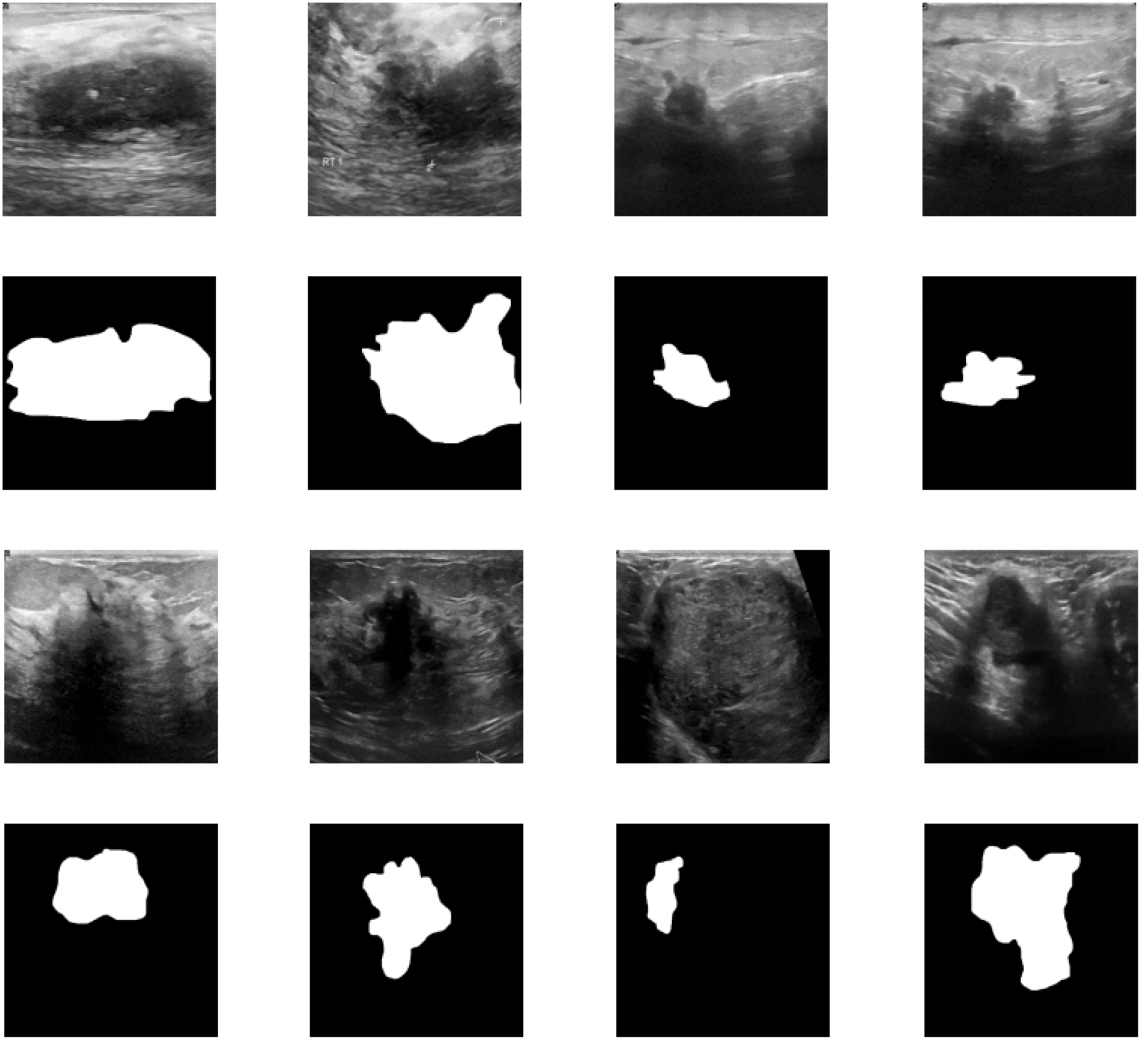
Segmentation results visualization.

**Figure 6:**
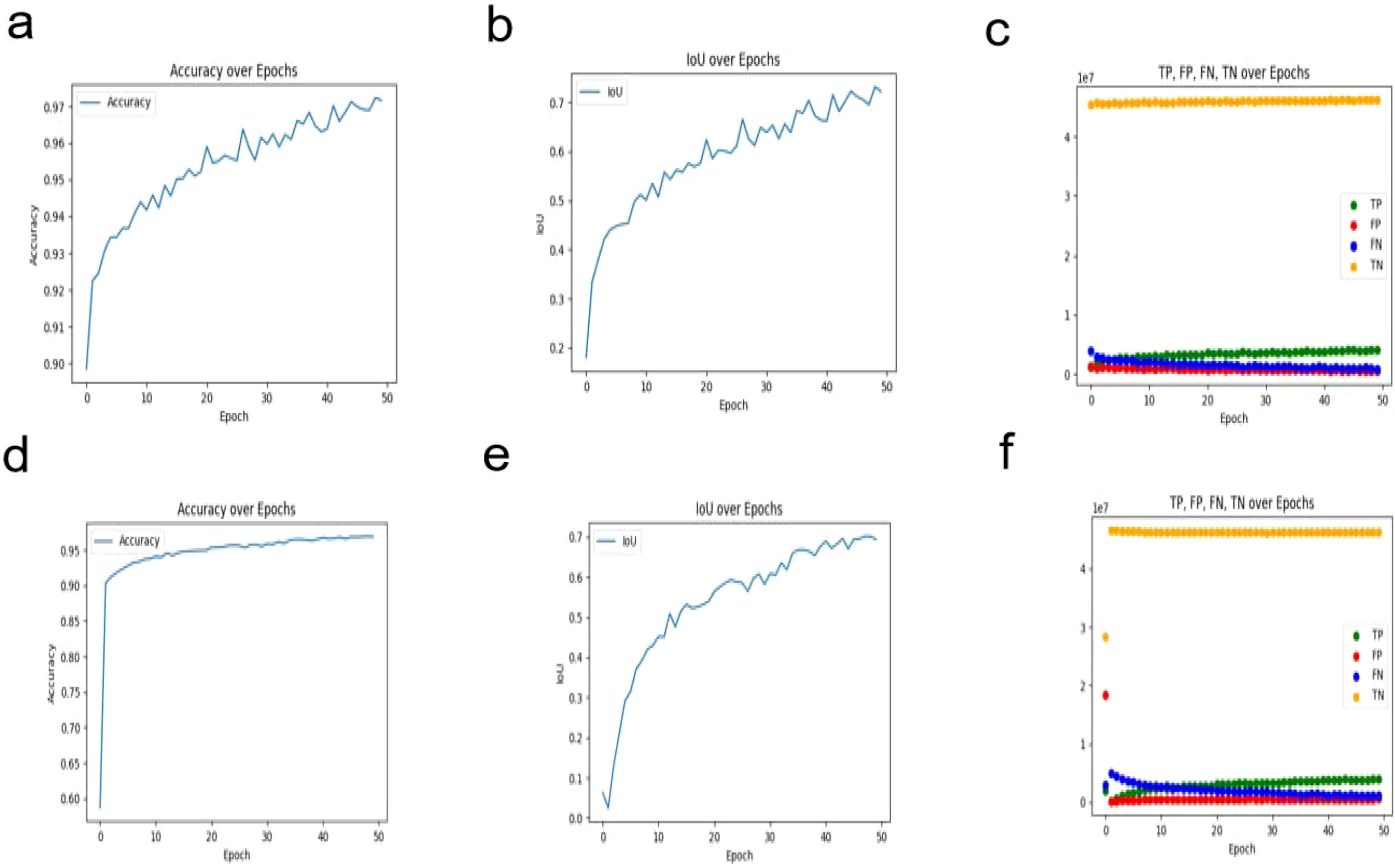
Segmentation Results Visualization. **(A)** U2NET-Lite Accuracy **(B)** U2NET-Lite Intersection over Union (IOU) **(C)** U2NET-Lite TP, FP, TN, FN **(D)** U2NET Accuracy **(E)** U2NET Intersection over Union (IOU) **(F)** U2NET TP, FP, TN, FN.

To visually demonstrate the model’s segmentation effectiveness, **Figures 5** and **6** include visualizations of segmented breast ultrasound images. These visual results clearly show the model’s accuracy and efficiency in identifying breast tumors and normal tissue. Additionally, **Table 1** presents the performance results of both U2NET-Lite and U2NET, highlighting their respective capabilities.

**Table 1:** Results of U2NET-Lite and U2NET.

Model	Accuracy	Recall	Precision	F1 Score	IoU
U2NET-Lite	0.9702	0.7961	0.7512	0.773	0.7063
U2NET	0.9686	0.7821	0.7379	0.7594	0.6938

### 3.2 Diagnostic model results

#### 3.2.1 Proposed model

**Figure 7:**
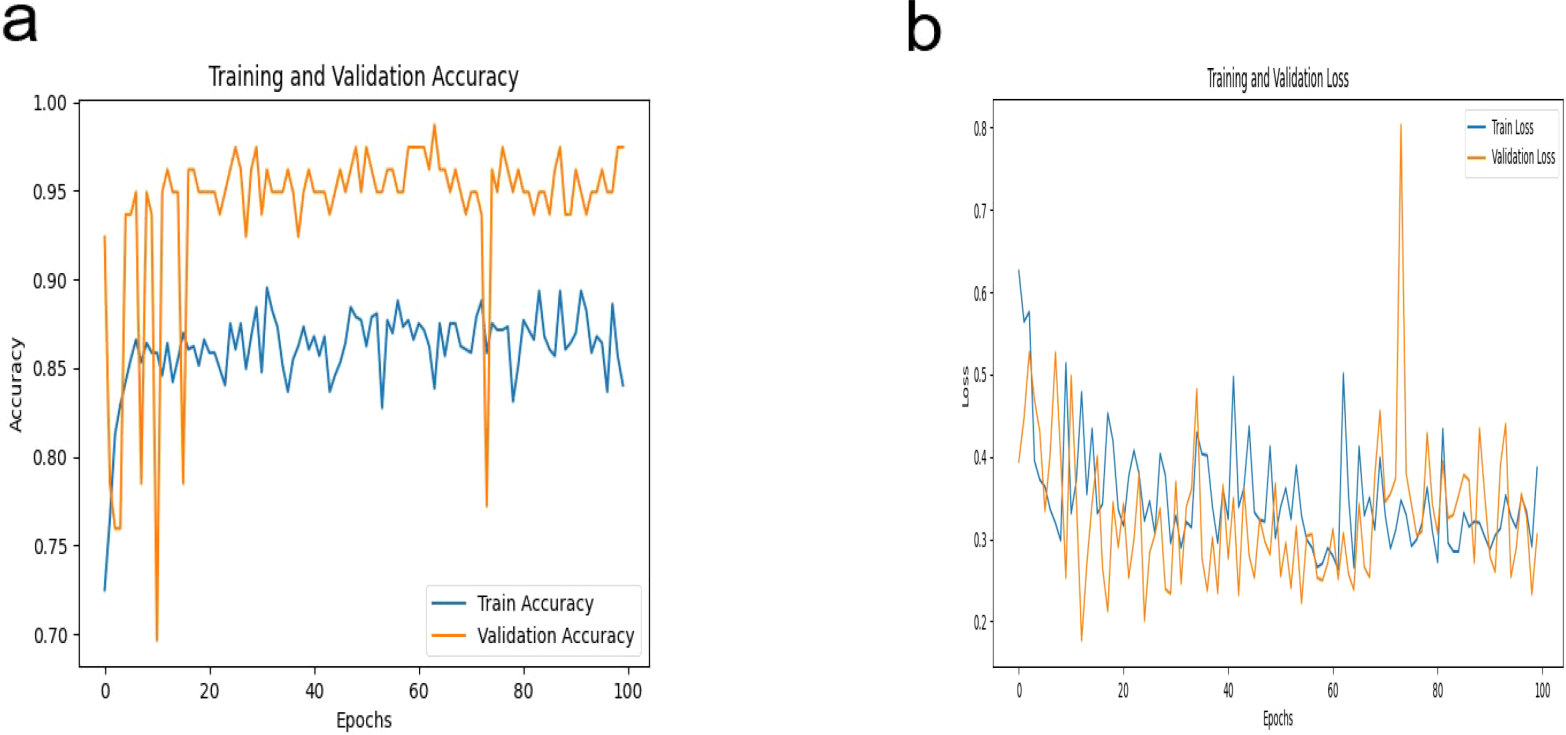
DeepCardinal-50 Model Training Iteration Diagram **(A)** Accuracy **(B)** Loss.

In this study, our proposed DeepCardinal-50 model exhibited outstanding performance in the automatic classification of breast tumors. During model training, we employed a pre-trained ResNet34 architecture, modifying the fully connected layer to accommodate binary classification output. We utilized the cross-entropy loss function and Adam optimizer with a learning rate of 0.0001 to ensure stable and efficient optimization over 100 epochs. **Figure 7** illustrates the training iterations of the DeepCardinal-50 model.

**Figure 8:**
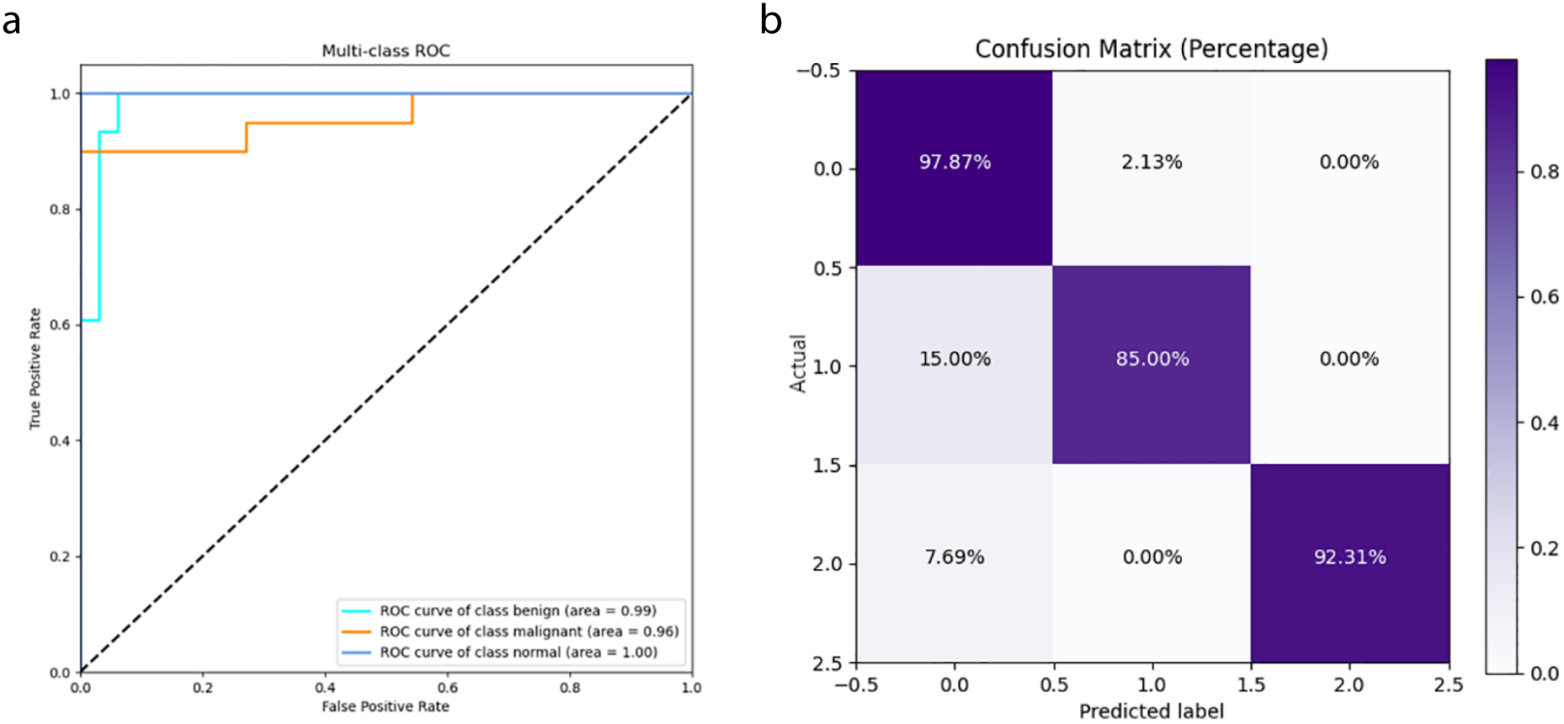
DeepCardinal-50 Model Training Results **(A)** ROC Curve **(B)** Confusion Matrix.

The DeepCardinal-50 model demonstrated excellent performance in the experimental test set. In the validation set, the model achieved a validation loss of 0.3052, a validation accuracy of 0.9747, a precision of 0.9548, a recall of 0.9173, and an F1 score of 0.9357. These results, shown in **Figure 8** and **Table 2**, reflect the model’s high accuracy, stability, and efficiency in the tasks of breast tumor identification and classification.

**Table 2:** Results of DeepCardinal-50 (best).

Model	Validation Loss	Validation Accuracy	Precision	Recall	F1 Score
DeepCardinal-50(best)	0.3052	0.9747	0.9548	0.9173	0.9357

#### 3.2.2 Comparative model results

**Figure 9:**
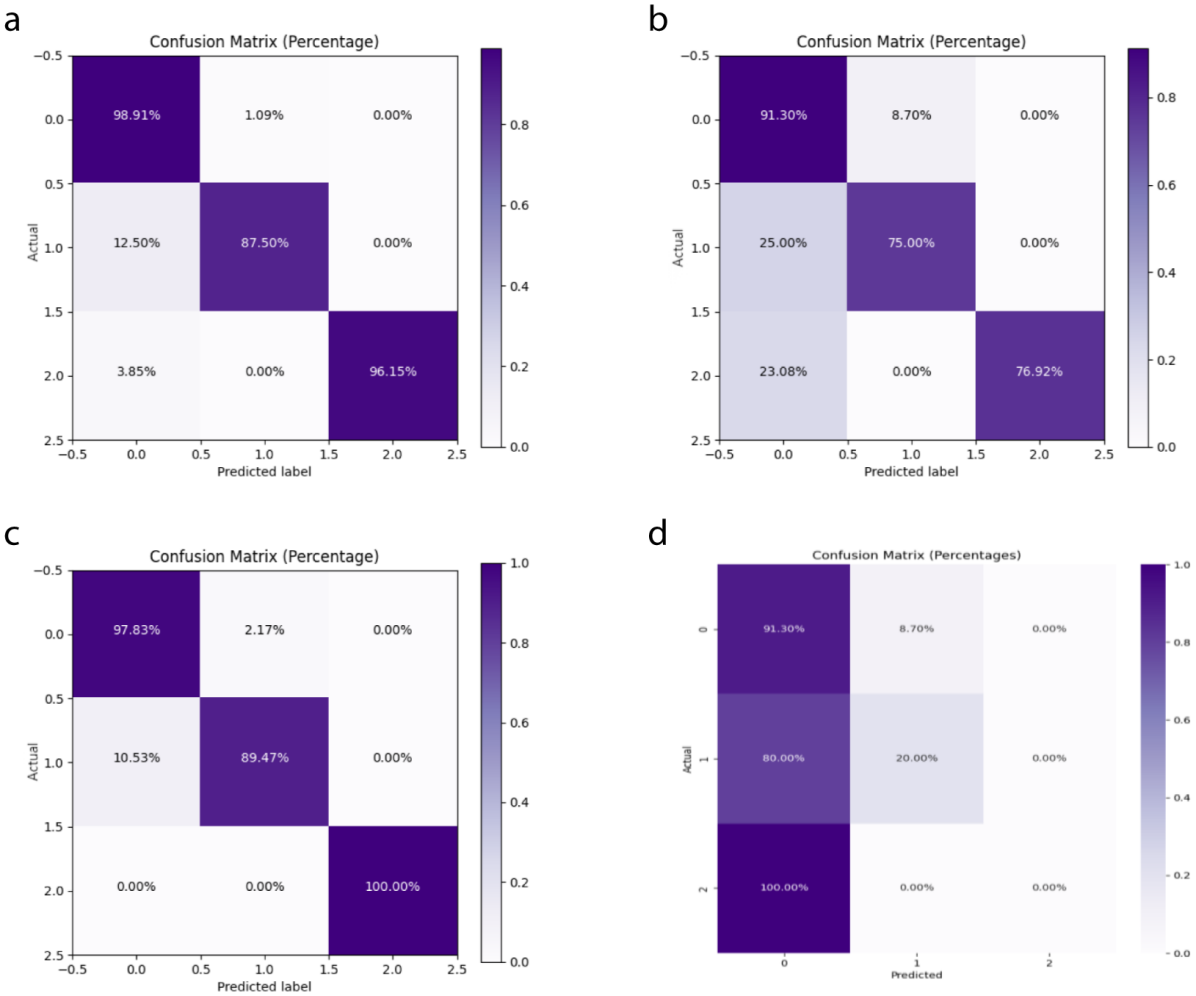
Ablation Study Confusion Matrices **(A)** ResNet34 with pre-training **(B)** ResNet34 **(C)** ResNet50 **(D)** AlexNet.

**Figure 10:**
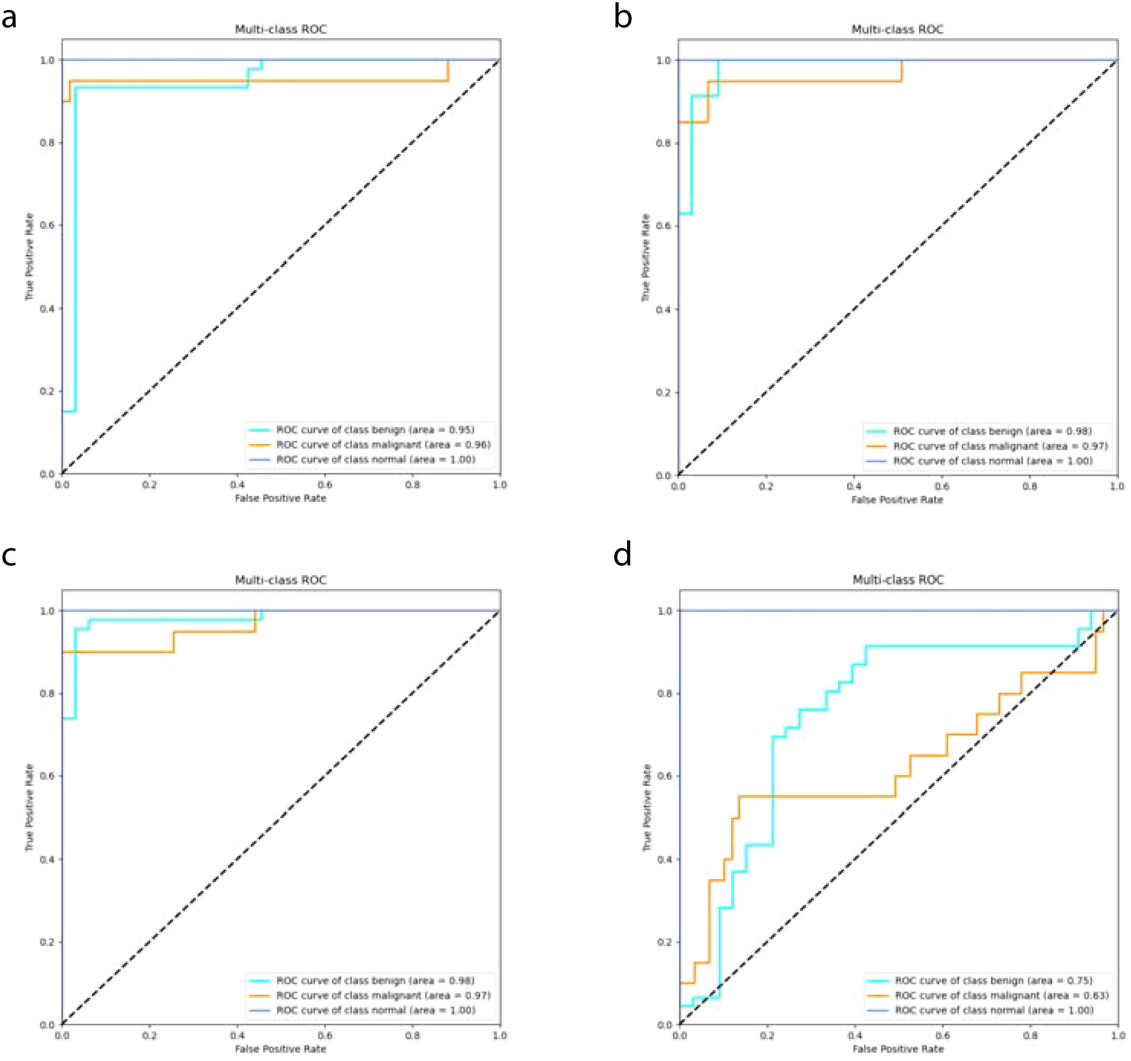
Ablation Study ROC Results **(A)** ResNet34 with pre-training **(B)** ResNet34 **(C)** ResNet50 **(D)** AlexNet.

In this study, we compared the DeepCardinal-50 model with other deep learning models and found that DeepCardinal-50 outperformed the others in the task of breast tumor classification. When compared with models such as ResNet-34, ResNet-50, and AlexNet, the DeepCardinal-50 exhibited superior performance across multiple metrics. The comparison results of the confusion experiments are shown in **Table 3**, the confusion matrix results of the ablation experiments are presented in **Figure 9**, and the detailed ROC results of the ablation experiments are illustrated in **Figure 10**.

**Table 3:** Comparison of ablation study results.

Model	Validation Loss	Validation Accuracy	Precision	Recall	F1 Score
DeepCardinal-50(best)	0.3052	0.9747	0.9548	0.9173	0.9357
ResNet50	0.2673	0.9747	0.9673	0.9577	0.9622
ResNet34	0.301	0.962	0.9701	0.9419	0.9548
ResNet34 with pre-training	0.2093	0.962	0.8765	0.8108	0.8379
AlexNet	0.7222	0.5823	0.3638	0.371	0.3346

In terms of validation loss, DeepCardinal-50 had a validation loss of 0.3052, whereas ResNet-50 and ResNet-34 had validation losses of 0.2673 and 0.301, respectively. Although ResNet-50 slightly outperformed DeepCardinal-50 in this metric, DeepCardinal-50 excelled in other critical metrics. For example, in validation accuracy, DeepCardinal-50 achieved 0.9747, while ResNet-50 also achieved 0.9747, and ResNet-34 achieved 0.962. Other models, such as ResNet-34 without pre-training and AlexNet, had validation accuracies of 0.962 and 0.5823, respectively, significantly lower than DeepCardinal-50.

DeepCardinal-50 also surpassed the comparative models in key metrics such as precision, recall, and F1 score. The DeepCardinal-50 achieved a precision of 0.9548, recall of 0.9173, and F1 score of 0.9357, while ResNet-50 achieved a precision of 0.9673, recall of 0.9577, and F1 score of 0.9622. AlexNet performed relatively poorly, with a precision of only 0.3638, recall of 0.371, and F1 score of 0.3346.

These comparative results indicate that DeepCardinal-50 exhibits outstanding performance in the task of breast tumor classification, with higher accuracy, precision, recall, and F1 score. These advantages make it an ideal choice for the automatic diagnosis of breast tumors, enabling clinicians to classify and diagnose breast tumors more effectively.

#### 3.2.3 Independent test results

**Figure 11:**
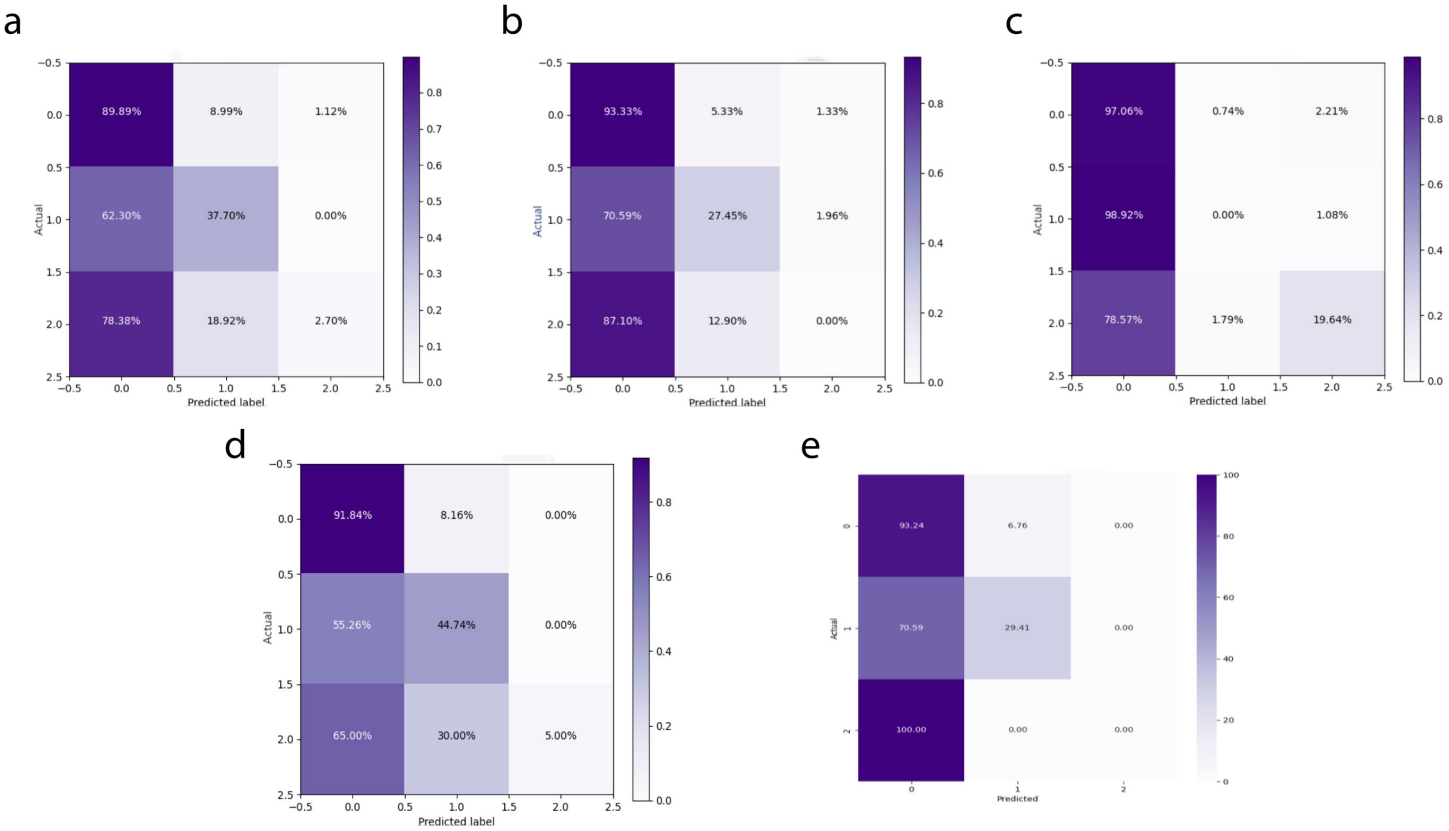
Independent Test Confusion Matrices **(A)** DeepCardinal-50 (best) **(B)** ResNet34 with pre-training **(C)** ResNet34 **(D)** ResNet50 **(E)** AlexNet.

**Figure 12:**
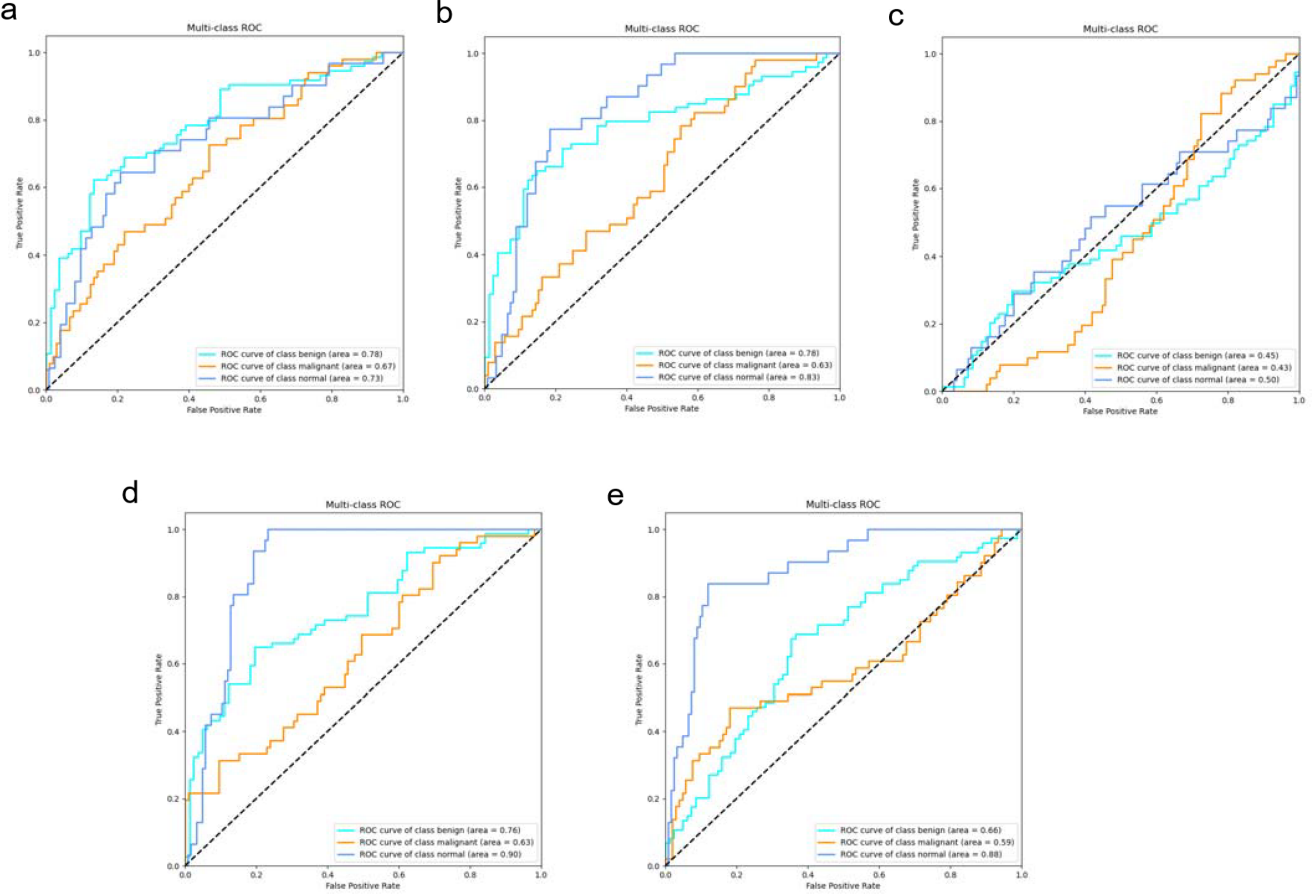
Independent Test ROC Curves **(A)** DeepCardinal-50 (best) **(B)** ResNet34 with pre-training **(C)** ResNet34 **(D)** ResNet50 **(E)** AlexNet.

In the independent testing phase of this study, we evaluated the performance of the DeepCardinal-50 model in breast tumor diagnosis and compared it with other models. The details are shown in **Table 4**, and the confusion matrix results for the independent testing are presented in **Figures 11** and **12**. The results showed that DeepCardinal-50 exhibited good performance across several key metrics, with a test loss of 2.1382 and a test accuracy of 0.5641. Although ResNet-50 had a lower test loss (1.3218) and a slightly higher test accuracy (0.609), DeepCardinal-50 excelled in other metrics. ResNet-34 and AlexNet had test accuracies of 0.4936 and 0.5385, and test losses of 2.3055 and 1.4921, respectively, which were notably inferior to DeepCardinal-50.

**Table 4:** Independent test comparison results.

Model	DeepCardinal-50	ResNet50	ResNet34 with pre-training	ResNet34	AlexNet
Test Loss	2.1382	1.3218	2.2287	2.3055	1.4921
Test Accuracy	0.5641	0.609	0.4936	0.4936	0.5385

To provide a clearer presentation of the test results, we included visualizations such as ROC curves and confusion matrices. The ROC curve for DeepCardinal-50 had a larger area, reflecting higher sensitivity and specificity in breast tumor diagnosis. The confusion matrix displayed the model’s prediction accuracy. These results indicate that DeepCardinal-50 demonstrates stability and reliability in the automatic diagnosis of breast tumors, showcasing significant clinical application value and broad prospects.

### 3.3 Clinical interpretability

**Figure 13:**
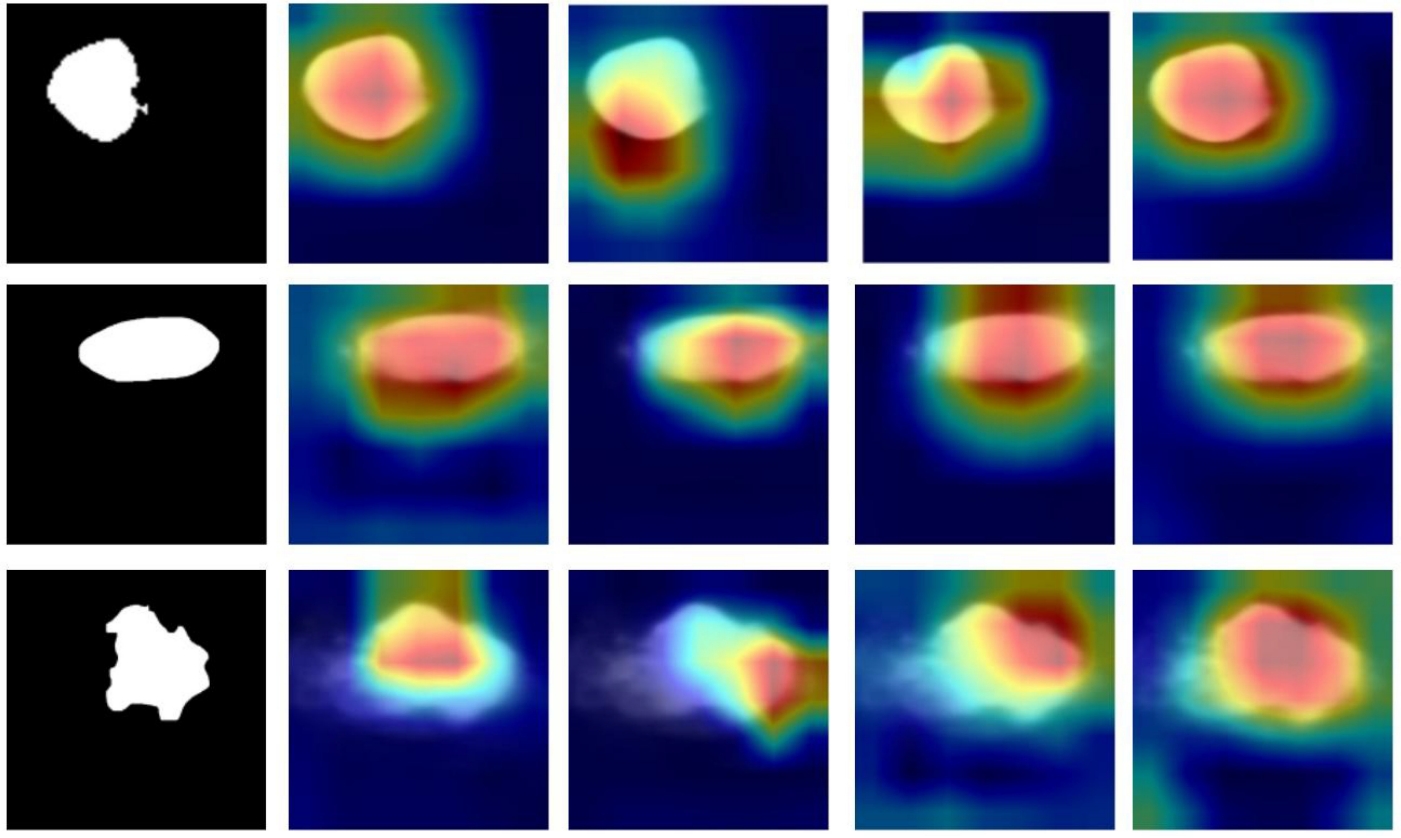
Heatmap results.

The machine learning models in this study, particularly U2NET-Lite and DeepCardinal-50, offer significant clinical interpretability for the ultrasound diagnosis of breast tumors. By analyzing ultrasound images, these models can accurately identify and classify breast tumors (16). More importantly, they can generate activation heatmaps that visually display the precise location and potential size of the tumors (17, 18) as shown in **Figure 13**. This visualization is crucial for physicians during the diagnostic process as it provides additional information about tumor characteristics, such as shape, edges, and the relationship with surrounding tissues (19, 20). This approach not only enhances the physician’s ability to determine the benign or malignant nature of breast tumors but also offers an intuitive way to understand the model’s decision-making process (21). Consequently, our method not only improves diagnostic accuracy and efficiency but also enhances the clinical applicability of ultrasound detection in breast tumor diagnosis (22, 23). This advancement is instrumental in promoting early detection and treatment of breast cancer, thereby contributing to better patient outcomes (24).

## 4 Discussion

This study leverages machine learning and ultrasonography to achieve intelligent and precise auxiliary diagnosis of breast tumors. We utilized the deep learning models DeepCardinal-50 and U2NET-Lite, which were specifically optimized for breast ultrasound imaging. Experimental results demonstrate that the optimized U2NET-Lite model exhibited high accuracy and recall rates on the test set, while the DeepCardinal-50 model achieved superior performance in classification tasks. The successful application of these models significantly improved diagnostic efficiency.

The optimal performance of our models is primarily attributed to their specialized architectural design and strong alignment with the task of breast tumor identification. From an architectural perspective, DeepCardinal-50 employs an enhanced 32x4d residual network structure, providing greater learning capacity and feature extraction precision, which is crucial for handling complex breast tumor images. U2NET-Lite, with its lightweight design, maintains core performance while adapting to the demands of real-time processing, which is particularly important for clinical applications. In terms of prediction tasks, these models effectively process high-resolution images and accurately identify tumor regions, which is critical for the early diagnosis of breast cancer. Therefore, the strong alignment between model architecture and the specific task of breast tumor identification is the key factor behind our outstanding results (25).

Compared with previous similar studies, Losurdo et al. regarded the automatic segmentation of breast DCE-MRI images aimed at overcoming the issue of background parenchymal enhancement (26), while Fanizzi et al. presented a CAD system capable of automatic detection of microcalcifications in digital mammographic images (27), exploiting the circular Hough transform. Lastly, Bove et al. provided an instance of a machine learning application to breast ultrasound images, combining clinical and radiomic features, that yielded promising results for the prediction of the sentinel lymph-node status through a non-invasive procedure (28). Similarly, the review by highlights the critical role of segmentation in CAD systems for breast tumor localization and detection, categorizing methods into supervised, unsupervised, and deep learning-based approaches. This comprehensive overview provides valuable insights for selecting suitable segmentation techniques based on specific clinical use cases, further emphasizing the importance of robust CAD methodologies for early cancer detection (29).

We have developed a new model that maintains high performance while reducing computational requirements, facilitating future dissemination. This work has the potential to extend beyond the identification of benign and malignant breast tumors and could be applied to other ultrasonography fields such as thyroid or liver cancer through dataset transformation (30).

However, this study has limitations. Firstly, the model currently only identifies benign and malignant types of breast tumors and can be further refined to include subtyping in the future. Additionally, our current research data is limited, and we need to use more multicenter data to further mature the model. Finally, this research has not yet been developed and deployed as a system, and further development is needed to achieve clinical translation.

## 5 Conclusion

In this research, we have successfully developed an intelligent assisted diagnosis system for breast tumors based on machine learning and deep learning, including a deep learning architecture based on segmentation and prediction of breast nodules as a whole. The system demonstrated high efficiency and accuracy in both classification and recognition of breast ultrasound images. These models provide a new technological pathway for early diagnosis and treatment of breast cancer. We will discuss our proposed models with ultrasound imaging specialists and physicians with a view to practical implementation in hospitals.

## Data Availability

The original contributions presented in the study are included in the article/supplementary material. Further inquiries can be directed to the corresponding author.
